# Activation-Induced Cytidine Deaminase Deficiency Causes Organ-Specific Autoimmune Disease

**DOI:** 10.1371/journal.pone.0003033

**Published:** 2008-08-21

**Authors:** Koji Hase, Daisuke Takahashi, Masashi Ebisawa, Sayaka Kawano, Kikuji Itoh, Hiroshi Ohno

**Affiliations:** 1 Research Center for Allergy and Immunology, RIKEN, Yokohama, Kanagawa, Japan; 2 Supramolecular Biology, International Graduate School of Arts and Sciences, Yokohama City University, Yokohama, Kanagawa, Japan; 3 Graduate School of Agricultural and Life Sciences, The University of Tokyo, Tokyo, Japan; Centre d'Immunologie de Marseille-Luminy, CNRS-Inserm, France

## Abstract

Activation-induced cytidine deaminase (AID) expressed by germinal center B cells is a central regulator of somatic hypermutation (SHM) and class switch recombination (CSR). Humans with *AID* mutations develop not only the autosomal recessive form of hyper-IgM syndrome (HIGM2) associated with B cell hyperplasia, but also autoimmune disorders by unknown mechanisms. We report here that AID^−/−^ mice spontaneously develop tertiary lymphoid organs (TLOs) in non-lymphoid tissues including the stomach at around 6 months of age. At a later stage, AID^−/−^ mice develop a severe gastritis characterized by loss of gastric glands and epithelial hyperplasia. The disease development was not attenuated even under germ-free (GF) conditions. Gastric autoantigen -specific serum IgM was elevated in AID^−/−^ mice, and the serum levels correlated with the gastritis pathological score. Adoptive transfer experiments suggest that autoimmune CD4^+^ T cells mediate gastritis development as terminal effector cells. These results suggest that abnormal B-cell expansion due to AID deficiency can drive B-cell autoimmunity, and in turn promote TLO formation, which ultimately leads to the propagation of organ-specific autoimmune effector CD4^+^ T cells. Thus, AID plays an important role in the containment of autoimmune diseases by negative regulation of autoreactive B cells.

## Introduction

The targeted deamination of Ig genes by AID is a prerequisite for Ab affinity maturation through somatic hypermutation (SHM) and class switch recombination (CSR) [1]. Therefore, AID deficiency leads to a defect in these two critical events in humoral immunity, and in humans causes hyper IgM syndrome HIGM2, a disease characterized by recurrent bacterial infections [2]. Enhanced proliferation of B cells and increased repertoire diversity were observed in aged AID^−/−^ mice, suggesting a critical role of AID in B-cell growth regulation [3]. AID^−/−^ mice also display abnormal expansion of anaerobic commensal bacteria in the small intestine, which induces hypertrophic enlargement of Peyer's patches and protrusion of isolated lymphoid follicles (ILFs). The abnormality of intestinal flora is due to the lack of hypermutated IgA, because reconstitution of intestinal IgA production recovered the normal composition of gut flora[4]. These results suggest that AID plays a key role in homeostasis of intestinal flora. Furthermore, a fraction of patients carrying *AID* mutations suffer from various organ-specific autoimmune diseases, including diabetes mellitus, autoimmune hepatitis and Crohn's disease, via unknown mechanisms [5].

In autoimmune-mediated tissue disorders, T cells are usually thought to be the most important cell type for controlling autoimmune responses. On the other hand, recent studies suggest that interactions between B and T cells play a pivotal role in the pathogenesis of autoimmue diseases [6]. The B-cell receptor (BCR) in developing B-cell precursors is produced via the rearrangement of randomly selected V, (D) and J segments of the Ig heavy and light chain loci. This Ig gene recombination is crucial to increase the diversity of the B-cell repertoire however, due to its stochastic nature, a substantial number of newly synthesized BCRs bind autoantigens. It was recently estimated that more than 50% of newly generated B cells are autoreactive [7]. Studies using transgenic mice carrying autoreactive BCR genes indicate that autoreactive B cells are normally silenced by immunological tolerance mechanisms including clonal deletion, receptor editing and anergy [8], [9]. However, in humans and mice that are prone to autoimmune diseases, the B-cell tolerance mechanisms seem to be overwhelmed by genetic or acquired defects. This concept is underscored by the finding that unregulated control of B-cell activation or proliferation due to the deficiency of the inhibitory Fcγ receptor (FcγRIIB), the protein phosphatase Shp1, or the protein kinase Cδ causes autoimmune diseases [10]–[12]. As a consequence, B-cell-targeting therapies have become one of the most effective treatments for autoimmue diseases [6], [13].

Although enhanced growth of B cells coupled with enlarged GC has been observed in AID*^−/−^* humans and mice, the potential contribution of AID to autoimmunity remains largely unknown. To better define the role of AID in autoimmunity, we carefully analyzed AID*^−/−^* mice at different ages. We found that aged mice spontaneously developed gastritis with pathological features similar to human type A gastritis and murine experimental autoimmune gastritis. The disease could be reconstituted in nu/nu mice by adoptive transfer of CD4^+^ T cells isolated from inflamed gastric tissue. Anti-gastric-mucosa-specific Ab were elevated in gastritis-positive, but not in gastritis-negative AID*^−/−^* mice, indicating that autoreactive B cells are propagated in the gastritis-positive AID*^−/−^* mice. Importantly, gastritis development was preceded by formation of tertiary lymphoid organs (TLO). Self-reactive B cells, likely generated because of increased BCR diversity in the absence of AID, in TLOs could capture large amounts of gastric autoantigens to present to T cells, and reciprocally receive T-cell help. Consequently, such a vicious cycle through a B-T collaboration could facilitate the generation of gastric antigen-specific CD4 T cells, which ultimately cause the pathology of autoimmune gastritis. The data presented here show that AID is necessary not only for B cell homeostasis but also for negative regulation of autoimmune disorders.

## Results

### Lymphoid neogenesis is frequently observed in AID^−/−^ mice

**Figure 1:**
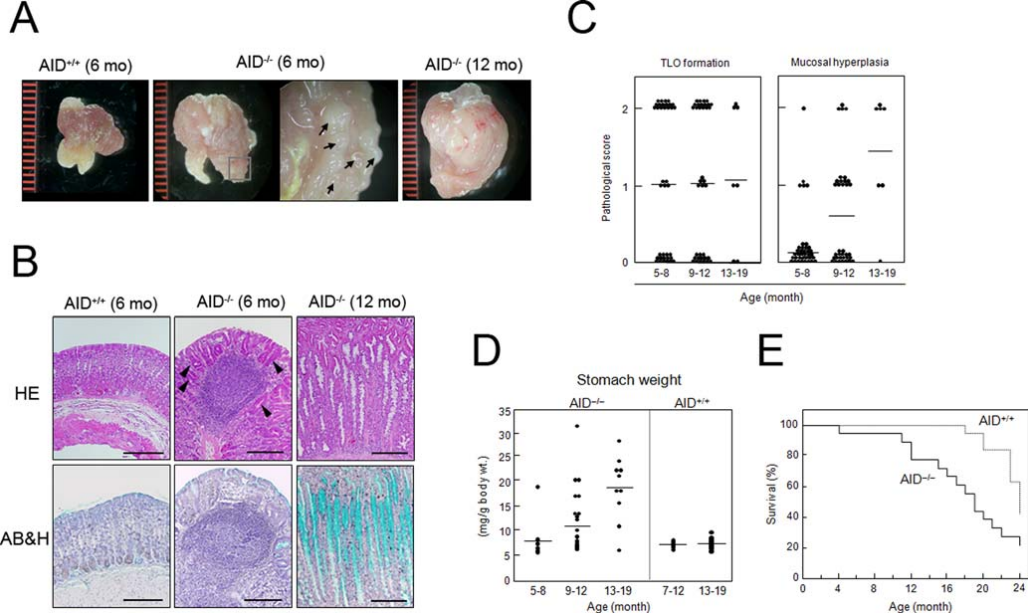
AID^−/−^ mice spontaneously developed gastritis. (A) Stomachs of AID^+/+^ and AID^−/−^ mice were cut longitudinally to expose the gastric mucosae. Inside-out tissues were examined by stereomicroscopy. A high magnification view of the mucosal surfaces revealed the presence of a number of nodule-like structures in the AID^−/−^ mouse stomach (arrows). A representative sample from each group is shown. (B) Gastric tissue sections were stained with H&E for histological examination, or with Alcian blue-hematoxylin (AB&H) for detection of mucin-producing cells (shown as turquoise). Scale bars: 100 µm. (C) Gastric tissues of AID^−/−^ mice at different ages were analyzed for development of ectopic lymphoid follicles and mucosal hyperplasia. The diagnostic criteria are described in Supplemental Table S1 online. (D) Stomach weights of AID^+/+^ and AID^−/−^ mice were measured and the values were normalized to the body weight of each mouse. (E) Kaplan-Meier survival curves of AID^−/−^ (solid line, n = 18) and AID^+/+^ (dotted line, n = 19) mice revealed significant differences in survival (*P*<0.05).

**Figure 2:**
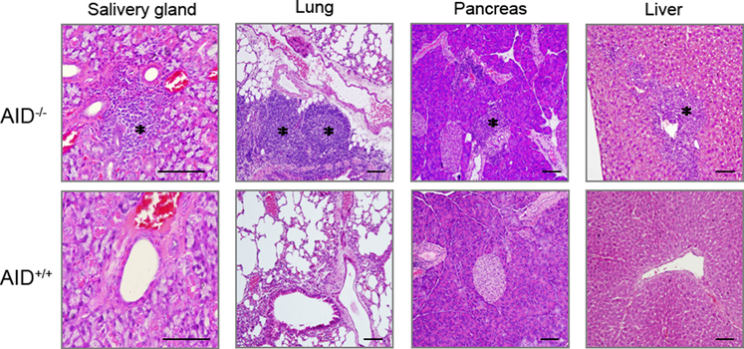
TLOs are formed in various organs of AID^−/−^ mice. The tissue samples were obtained from 12-month-old AID^−/−^ or AID^+/+^ mice, and were subjected to histological examination after H–E staining. Asterisks represent TLOs. Scale bars: 100 µm.

**Figure 3:**
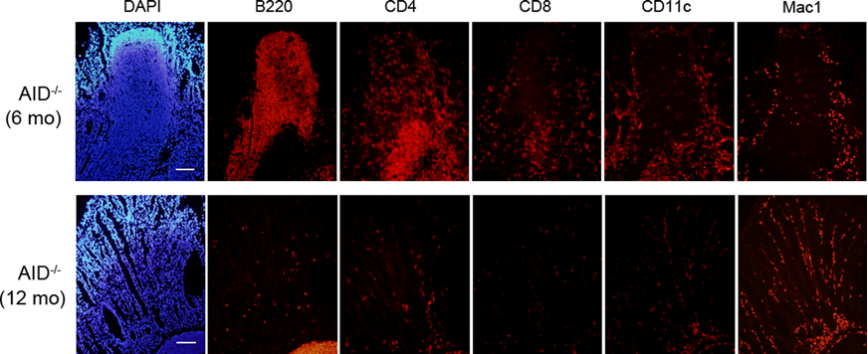
Massive infiltration of inflammatory cells is observed in the gastric tissue of AID^−/−^ mice. Immunofluorescence staining for immune cell-specific markers was performed using serial tissue sections prepared from two different gastric samples with ectopic lymphoid follicles (upper) or mucosal hyperplasia (lower). DAPI staining (left panels) was used to identify cell nuclei. Scale bars: 100 µm.

We observed that AID^−/−^ mice housed under specific pathogen-free (SPF) conditions developed a number of polyp-like lesions in the gastric mucosa (Figure 1A, see arrows). Histological examination revealed that these lesions resulted from the formation of TLOs (Figure 1B, central panels). Multifocal lymphoid structures developed in the stomachs of 70% of AID^−/−^ mice at 5–8 months (Figure 1C). Similar lymphoid neogenesis was detected in other organs such as lung, salivary gland, liver and pancreas in AID^−/−^ mice (Figure 2), albeit at lower frequencies. Analogous to secondary lymphoid tissues, gastric TLOs were composed of segregated B-cell and T-cell areas (Figure 3, upper panels). Furthermore, GCs, characterized by CXCL13 and CR-1 expression, were observed in the B-cell follicles of TLOs (Figure S1A, left column). Of note is that IgM^hi^B220^lo^ plasma cells were scattered in the lamina propria surrounding the TLOs (Figure S1A, right column). H&E staining of gastric TLOs further confirmed the presence of plasma cells with the characteristic appearance of nuclei and abundant cytoplasm in the circumference of the TLOs (Figure S1B, see arrows). These observations suggest the possibility that B cells activated in the gastric TLO would differentiate and directly migrate from the lymphoid follicles to the gastric lamina propria without passing through the systemic circulation.

### Aged AID^−/−^ mice spontaneously develop gastritis

A reduction of parietal cells was evident around the circumference of TLOs (Figure 1B, see arrowheads). This pathological change was remarkably increased in older mice. The stomach of AID^−/−^ mice around 12 months old was greatly enlarged (Figure 1A and D). Histological analysis of the enlarged stomachs revealed epithelial hyperplasia and destruction of gastric glands (Figure 1B and C) coupled with an increase in mucus-producing cells (Figure 1B, lower panels). Vestigial TLOs were frequently associated with the hyperplastic gastric lesions (Figure S1C). A massive infiltration of inflammatory cells was also detected in the lamina propria of the gastric tissue of AID^−/−^ mice. Immunofluorescent staining revealed that these infiltrating cells were mainly Mac1/CD11b^+^ cells (Figure 3, lower panels). Other subsets of immune cells, namely, B cells, CD4^+^ and CD8^+^ T cells, and CD11c^+^ cells were also found scattered in the inflamed tissue. In contrast, neither age-matched AID^+/+^ (Figure 1A) nor AID^+/−^ mice (data not shown) developed TLOs or gastritis, although minimal lymphocyte infiltration was detected in the gastric tissues of occasional mice in these control groups (Figure S2, and data not shown). Perhaps associated with increasingly severe gastritis development, more than 20% of AID^−/−^ mice died by 12 months of age, and half of them died by 18 months, whereas almost all AID^+/+^ mice survived for more than 18 months (Figure 1E, *P*<0.05).

### Cellular phenotype of gastric mucosa-infiltrating lymphocytes in AID^−/−^ mice

**Figure 4:**
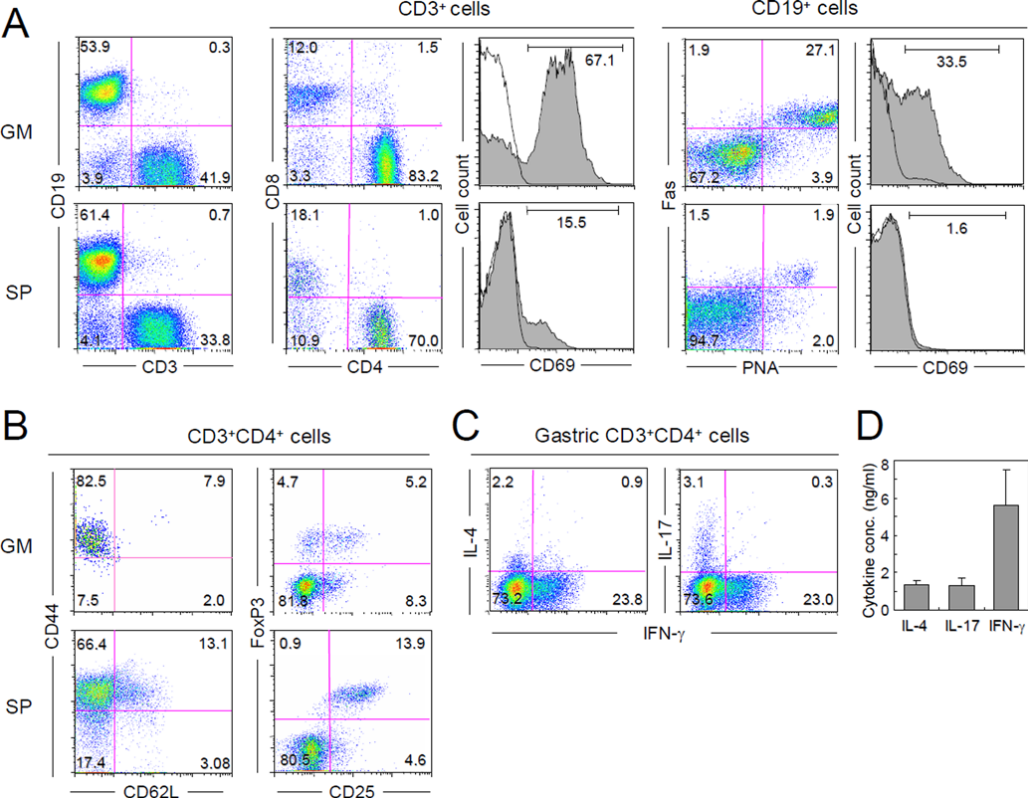
Gastric mucosa-infiltrating lymphocytes in AID^−/−^ mice have an activated phenotype. (A) Lymphocytes were prepared from gastric mucosae (GM; upper panels) of AID^−/−^ mice with gastritis. Since lymphocyte infiltration into gastric tissues was negligible in AID^+/+^ mice (Figure 1B), splenocytes (SP; lower panels) from AID^−/−^ mice with gastritis were used as a control. (B) CD3^+^CD4^+^ gastric and splenic lymphocytes were gated and further analyzed for memory and regulatory T-cell markers. (C) Gastric CD3^+^CD4^+^ cells enriched with a MACS column were treated with PMA and Golgi-stop for 6 h and then analyzed for production of the indicated cytokines by intracellular staining. (D) Concentrations of the indicated cytokines in culture supernatants of gastric CD4^+^ cells stimulated with anti-CD3 and anti-CD28 mAbs for 48 h were determined by CBA and ELISA.

To further characterize the immunopathology of the gastritis, we first performed FACS analysis of gastric mucosa-infiltrating lymphocytes in AID^−/−^ mice. As shown in Figure 4A, gastric B and T cells highly upregulated the activation marker CD69, in comparison to splenic lymphocytes. The percentage of GC B cells, characterized by Fas expression and peanut agglutinin (PNA) binding, was much higher in the stomach (21.2±8.6%, n = 5) than in the spleen (2.2±0.9%, n = 3; *P*<0.01) (Figure 4A). These data indicate that active immune responses take place in the gastric tissues of AID^−/−^ mice. Additionally, most gastric mucosa-infiltrating CD4^+^ T cells had a CD44^hi^CD62L^lo^ effector/memory phenotype (Figure 4B, left panels). The ratio of FoxP3^+^ Treg cells to total CD4^+^ T cells was significantly lower in gastritis lesions (12.6±1.7%, n = 5) than in the spleen (19.0±3.8%, n = 3; *P*<0.01). Interestingly, the surface expression level of CD25 in gastric FoxP3^+^CD4^+^ T cells was somewhat lower than that on splenic FoxP3^+^CD4^+^ T cells (Figure 4B, right panels), and nearly half of FoxP3^+^ CD4^+^ T cell were even negative for CD25 expression.

### Proinflammatory cytokines are upregulated in the stomach of AID^−/−^ mice

**Figure 5:**
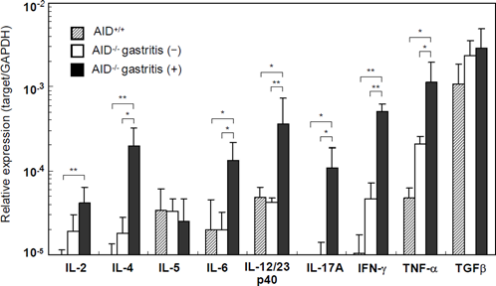
Q-PCR analysis of cytokine mRNA expression in gastric tissue. Gastric tissue samples were obtained from 12-month-old AID^+/+^ mice and AID^−/−^ mice with or without gastritis symptoms. Data are means±S.D. of at least four different samples. *P<0.05; **P<0.01.

We subsequently measured cytokine expression profiles in the gastric tissue. Quantitative real-time PCR (Q-PCR) analysis demonstrated that proinflammatory cytokines TNF-α and IL-12/23p40 were highly upregulated in gastritis-positive AID^−/−^ mice, but not in AID^+/+^ or gastritis-negative AID^−/−^ mice (Figure 5). Additionally, Th1 (IFN-γ), Th2 (IL-4) and Th17 (IL-17) cytokines were significantly upregulated in the inflamed gastric tissue. Likewise, intercellular FACS staining and ELISA data confirmed that a substantial number of IL-4-, IFN-γ- and IL-17-producing CD4^+^ T cells were present in gastric-mucosa-infiltrating cells (Figure 4C and D).

### The gastrointestinal microflora are irrelevant to the etiology of lymphoid neogenesis and gastritis in AID^−/−^ mice

The data obtained so far indicate that AID^−/−^ mice spontaneously develop lymphoid neogenesis followed by gastritis with mucosal hyperplasia. However, it is still unknown how AID deficiency leads to the development of gastritis. Fagarasan et al. previously reported that AID deficiency leads to aberrant expansion of anaerobic bacteria in the gut, which induces hypertrophy of Peyer's patches and ILFs as well as activation of the systemic immune system [3]. This observation raised the possibility that activation of AID^−/−^ B cells by gastric microflora might elicit lymphoid neogenesis followed by gastric tissue destruction. Similar pathological mechanisms have been reported in *Helicobacter pylori*-induced gastritis and MALT lymphoma, diseases in which the eradication of *H. pylori* cured most cases [14], [15]. Therefore, to explore a role of gastric microflora in gastritis development, we maintained AID^−/−^ mice in either GF or conventional (CV) conditions, and examined gastritis development. If an unregulated response of AID^−/−^ B cells to gastric microflora was involved in the pathogenesis of gastritis, the GF environment should prevent gastritis development and, conversely, CV conditions might worsen the disease compared to that seen under SPF conditions.

**Figure 6:**
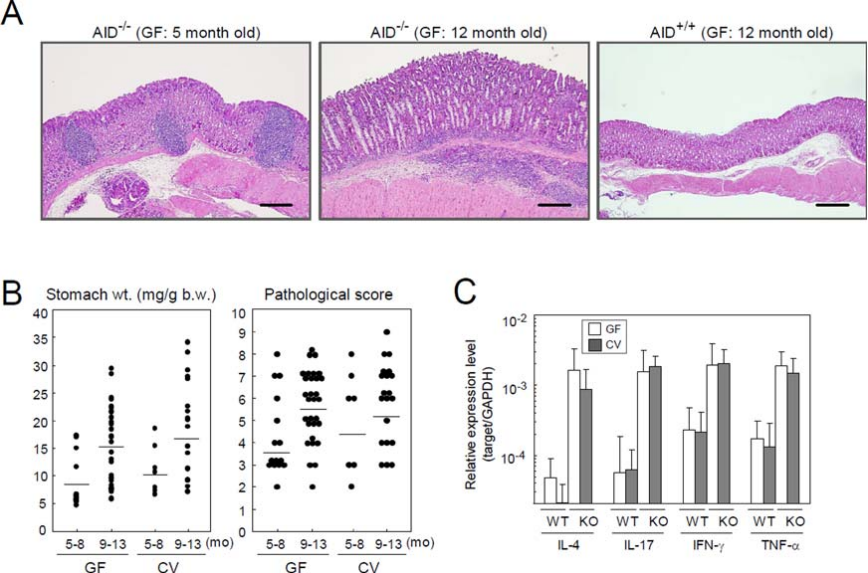
Gastric microflora are irrelevant to gastritis development in AID^−/−^ mice. AID^−/−^ and AID^+/+^ mice were raised under germ-free (GF) or conventional (CV) conditions to examine whether the presence of gastric microflora affects the development of gastritis. (A) Histological examination of gastric tissues from AID^−/−^ and AID^+/+^ mice maintained under GF condition. (B) Stomach weight and pathological score of gastritis were compared between AID^−/−^ mice in GF and CV conditions. (C) Cytokine expression levels in gastric tissues of AID^−/−^ and AID^+/+^ mice under GF and CV conditions were measured by Q-PCR. The mRNA expression level of each cytokine was normalized to that of GAPDH.

The hypertrophic protrusion of ILFs was not observed in AID^−/−^ mice maintained at GF condition (data not shown), as expected by the previous report with antibiotic cocktail treatment [4]. By contrast, even under GF conditions, AID^−/−^ mice developed TLOs at 5 months of age, and subsequently gastritis lesions with epithelial hyperplasia and massive inflammatory cell infiltration at 12 months (Figure 6A). There was no difference in stomach weight, pathological score, or cytokine expression between AID^−/−^ mice housed under GF and CV conditions (Figure 6B and C), suggesting that the gastric microflora are irrelevant to the pathogenesis of gastritis.

### Gastric Ag-specific autoreactive B cells are generated in AID^−/−^ mice

**Figure 7:**
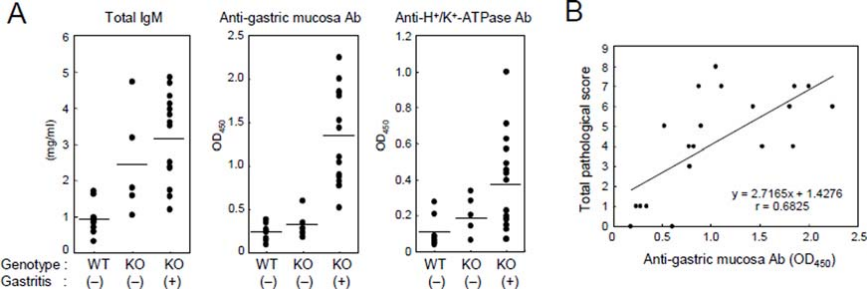
Gastric mucosa-specific antibodies are elevated in AID^−/−^ mice. (A) Serum samples were collected from AID^+/+^ and AID^−/−^ mice maintained under SPF condition to measure total IgM, and gastric-mucosa- or H^+^/K^+^-ATPase-specific IgM levels by ELISA. After histological examination, individuals with a total pathological score of 2 or more were considered to be gastritis-positive. (B) Serum levels of anti-gastric mucosa antibodies positively correlated with total pathological score in AID^−/−^ mice (r = 0.6825; *P*<0.05).

It has been reported that AID^−/−^ B cells are constitutively activated without immunization, and that their germline-encoded BCR diversity is dramatically increased compared to wild-type mice [3]. We therefore hypothesized that such an aberrant diversification of the B-cell repertoire might produce abundant autoreactive B cells that eventually would overwhelm peripheral tolerance mechanisms. To test this hypothesis, we measured serum levels of total and gastric mucosa-specific IgM in AID^−/−^ and AID^+/+^ mice. As expected [1], the total IgM levels were higher in AID^−/−^ mice than in wild type mice whereas they were comparable between gastritis-negative and -positive AID^−/−^ mice (Figure 7A). By contrast, the serum levels of gastric mucosa-specific Ab were significantly higher in gastritis-positive than in gastritis-negative AID^−/−^ mice. Moreover, there was a positive correlation between serum autoantibody levels and pathological gastritis scores in AID^−/−^ mice (Figure 7B). H^+^/K^+^ ATPase could be one of the self antigens involved in this response, since the serum levels of anti-H^+^/K^+^ ATPase antibodies tended to be elevated in gastritis-positive AID^−/−^ mice (Figure 7A). These observations confirmed that B-cell clones specific for gastric autoantigens are generated in AID^−/−^ mice.

### Organ-specific autoreactive CD4^+^ T cells act as terminal effectors in the development of gastritis

**Figure 8:**
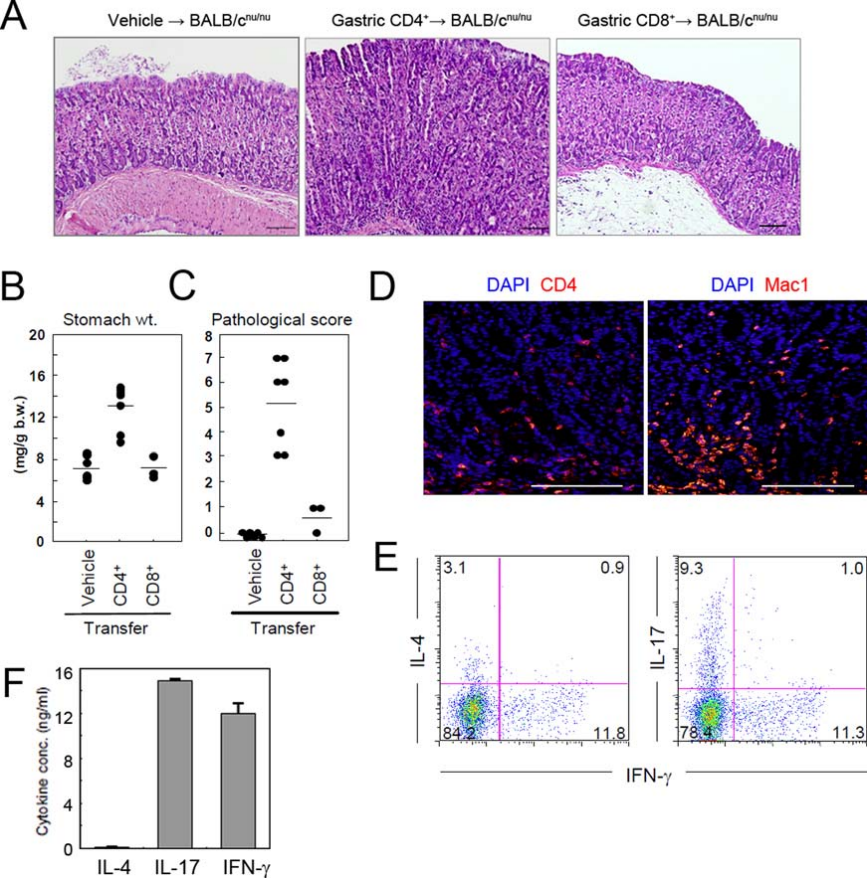
CD4^+^ T cells contribute to the development of gastritis in AID^−/−^ mice. Gastric mucosa-infiltrating CD4^+^ T cells or CD8^+^ T cells were isolated from 12 month old AID^−/−^ mice and were adoptively transferred into BALB/c^nu/nu^ mice. After 3 months, the stomachs of recipient mice were removed and subjected to the following experiments. (A) Gastric tissue sections were stained with H&E for histological examination. (B) Stomach weights were normalized to body weight of each mouse. (C) Gastric tissue sections were examined by histology and assigned a pathological score. (D) Immunofluorescence staining revealed a massive infiltration of CD4^+^ and Mac1^+^ cells into the stomachs of BALB/c^nu/nu^ mice that received CD4^+^ T cells by adoptive transfer. (E) Gastric CD3^+^CD4^+^ cells enriched with a MACS column were treated with PMA and Golgi-stop for 6 hr and analyzed for cytokine production by intracellular staining. (F) CD4^+^ T cells recovered from gastric tissues of CD4^+^ T-cell-transferred BALB/c^nu/nu^ mice were stimulated with anti-CD3 and anti-CD28 mAbs for 48 h. Cytokine concentrations in the culture supernatants were measured by CBA and ELISA. Scale bars: 100 µm.

The data obtained by FACS analysis revealed that gastric mucosa-infiltrating CD4^+^ T cells were activated and produced large amounts of multiple cytokines (Figure 4C). These data suggest that CD4^+^ T cells may participate in gastritis development in AID^−/−^ mice. To test this possibility, we isolated gastric mucosa-infiltrating CD4^+^ and CD8^+^ T cells from AID^−/−^ mice and adoptively transferred them to BALB/c^nu/nu^ mice that lack T cells. Three months later, mice that received CD4^+^, but not CD8^+^, T cells developed gastritis characterized by a massive infiltration of Mac1^+^ cells, epithelial hyperplasia and reduction of gastric glands (Figure 8A–D). This phenotype was quite similar to that observed in the late stages of gastritis in AID^−/−^ mice (Figure 1B and Figure 3, lower panels), except for the absence of TLO. Furthermore, gastric CD4^+^ T cells recovered from BALB/c^nu/nu^ mice produced significant quantities of IL-17 or IFN-γ but not IL-4 (Figure 8E and F), suggesting that Th1 and/or Th17 cells function as terminal effectors of gastritis development in AID^−/−^ mice.

## Discussion

In the present study we have shown that as they age, AID^−/−^ mice sequentially develop lymphoid neogenesis followed by severe gastritis. Consequently, AID^−/−^ mice had a significantly shorter lifespan compared to wild type mice. Although AID deficiency was reported to lead to aberrant expansion of anaerobic bacteria in the gut, we found that gastritis development in AID^−/−^ mice results from an autoimmune reaction rather than hyper responsiveness to gastric microflora. This view is supported by several observations. First, even under GF conditions, both lymphoid neogenesis and gastritis occurred in the AID^−/−^ mice , whereas hypertrophy of ILFs was completely prevented (Hase K., unpublished observation). This result clearly suggests that lymphoid neogenesis in the stomach and hypertrophy of ILFs in the small intestine are induced by the different mechanisms in AID^−/−^ mice, although they share a similar appearance. Second, gastric mucosa-specific autoantibody was detected in gastritis-positive AID^−/−^ mice, and there was a positive correlation between serum autoantibody levels and pathological gastritis scores. Detection of autoantibody indicates the activation and differentiation of gastric antigen-specific B cell clones. Third, CD4^+^ effector T cells, which are likely autoreactive T cells typically observed in organ-specific autoimmune diseases, are generated in AID^−/−^ mice. These results imply an important role of AID for the containment of autoimmunity.

The gastritis that developed in aged AID^−/−^ mice was characterized by a massive infiltration of Mac1/CD11b^+^ cells and the destruction of parietal cells. The gastric glands in these mice were replaced by mucous-producing cells, resulting in epithelial hyperplasia. All of these pathological features are reminiscent of human type A gastritis [16] and its experimental model, murine autoimmune gastritis, which are well-characterized organ-specific autoimmune diseases. Experimental autoimmune gastritis can be induced in BALB/c mice subjected to thymectomy 3 days after birth [17], [18]. Approximately 60% of neonatally thymecomized mice develop gastritis within 12 weeks. In this model, CD4^+^ T cells but not CD8^+^ T cells prepared from the gastric mucosa can transfer the disease to nu/nu mice, indicating an essential role of CD4^+^ T cell in disease progression. Similarly, the gastritis that spontaneously develops in AID^−/−^ mice at later stages could be reconstituted in nu/nu mice by adoptive transfer of CD4^+^ T cells. This observation further confirms that AID^−/−^ mice spontaneously develop autoimmue gastritis through generation of gastric antigen-specific autoreactive T cells.

Of prime importance, lymphoid neogenesis precedes gastritis development in AID^−/−^ mice (Figure 1A–C). Furthermore, indications of gastritis lesions, such as reduction in number of parietal cells, were more obvious at the circumference of TLOs both at early (Figure 1B, see arrowheads) and late stages (Figure S1C). These observations indicate that gastritis occurs secondary to TLO formation in AID^−/−^ mice. Although lymphoid neogenesis has been detected in various organ-specific autoimmune disorders, including Hashimoto's thyroiditis, rheumatoid arthritis, type 1 diabetes and autoimmune gastritis [19]–[24], the mechanisms leading to lymphoid neogenesis as well as its immunopathological roles in autoimmune diseases are not fully elucidated. Given that TLO are apparently equipped with the complete set of lymphoid structures, i.e., secondary B-cell follicles with GC and distinct T-cell areas containing dendritic cells and high endothelial venules [25], TLO may play a role in maintaining an autoimmune response against persistent self antigens. This concept is at least party supported by experimental studies using the rat insulin promoter-lymphocytic choriomeningitis virus glycoprotein (RIP-GP) transgenic mouse model, where a positive correlation between lymphoid neogenesis and development of autoimmune diabetes was reported [21]. Furthermore, expression of the recombination-activating genes RAG1 and RAG2 is evident in ectopic GC formed in rheumatoid arthritis and autoimmune thyroid diseases [19], [26], [27]. RAG genes, which are normally expressed in primary lymphoid organs, are master regulators of the rearrangement of V(D)J gene segments. The induction of RAG genes in TLOs raises the possibility that secondary BCR rearrangements, termed receptor revision, occur at these sites and may contribute to the generation of autoreactive B cells. In parallel, a recent study indicated that B-cell specificities in pancreatic TLO of NOD mice are skewed to enhance autoantigen-binding capacity, suggesting that TLO promote selection of autoreactive B cells [20]. Once autoreactive B cells are selected and enhanced in TLO, these cells could differentiate into autoantibody-forming plasma cells (Figure S1A and B). Consistent with this idea, there is a positive correlation between serum autoantibody levels and numbers of TLO in several autoimmune disorders [19], [24]. However, in the case of AID^−/−^ mice, B cells can produce only low-affinity IgM due to the lack of SHM and CSR. Therefore, gastric antigen-specific autoantibodies would be expected to only minimally contribute to the pathogenesis of the gastritis. Instead, the more important mechanism by which autoreactive B cells promote inflammatory processes may be T-cell activation through presentation of autoantigens and co-stimulation. It has been suggested that autoreactive B cells have a crucial role as APCs to capture and present autoantigens for activating T cells, which in turn drive T-cell autoreactivity [28], [29]. This scenario could indeed be the case in the pathogenesis of autoimmune gastritis in AID^−/−^ mice. Autoimmune B cells could be selected and propagated in TLO formed in the stomach of AID^−/−^ mice in the early stages. Given that B cells in gastric TLO are in a privileged location to capture large amount of autoantigens and present them to T cells, this interaction could contribute to the pathogenesis by induction of autoreactive CD4^+^ T cells.

It should be noted that TLOs were induced not only in the stomach but also in other organs of AID^−/−^ mice (Figure 2). Therefore, these organs are also considered to be potential targets for an autoimmune response. Indeed, autoimmune diseases have been found to develop in multiple organs in humans with *AID* mutation [5]. The precise mechanism by which AID deficiency promotes the generation of self-reactive B cells is an open question at this moment. A previous report revealed that the BCR diversity is increased in AID^−/−^ mice [3]. Consequently, gastric Ag-specific B cells might emerge from the expanded B-cell repertoire. Under physiological conditions, B-cell autoimmunity is counteracted by several tolerance mechanisms including receptor editing, anergy, and clonal deletion. Particularly, as many as 50% of newly produced B cells are reported to show an anergic phenotype (CD93^+^B220^+^IgM^lo^), suggesting the primary importance of anergy in peripheral tolerance [30]. Although anergic B cells have a shorter lifespan relative to wild-type mature B cells [31], anergy is a reversible process: anergic B cells have been shown to revert to naïve B cells upon hapten stimulation [32]. This observation raises the possibility that escape from anergy could result in autoimmunity. Mice and humans lacking functional AID display B-cell hyperplasia and a hyperactivated immune system [1]–[3]. Based on these observations, we speculate that the aberrant activation stress may drive B-cell clones, which are normally retained in an anergic state, toward an autoimmune response. Intercrossing of AID ^−/−^ mice with transgenic mice expressing a BCR prone to anergy should help clarify this speculation.

Several mechanisms have been proposed to explain why AID deficiency leads to dysregulated proliferation of B cells. One possibility is that AID^−/−^ B cells are devoid of inhibitory signals though FcγRIIB due to the lack of IgG Ab production. FcγRIIB is the only FcγR that contains a cytoplasmic ITIM motif. Crosslinking of the BCR and FcγRIIB by IgG antibody-antigen complexes leads to negative feedback regulation for B cell activation via the phosphorylation of ITIM tyrosines and subsequent recruitment of the inositol phosphatase SHIP to the plasma membrane. Therefore, FcγRIIB^−/−^ mice display elevated Ig levels and enhanced immune complex-mediated tissue injury in response to Ag challenge [33]. Furthermore, involvement of this inhibitory receptor in peripheral tolerance has been supported by the fact that FcγRIIB^−/−^ mice on a C57BL/6 background spontaneously develop autoantibody-mediated lupus glomerulonephritis [10]. Although significantly different types of autoimmue diseases, namely systemic versus organ-specific, are induced in FcγRIIB^−/−^ and in AID^−/−^ mice, respectively, this phenotypic difference is most likely due to the inability of AID^−/−^ mice to produce high-affinity autoimmune IgG Abs. AID^-/-^ mice produce abnormally large amounts of IgM, whereas IgG is absent. IgM, like IgG, can promote complement activation, which in turn could support B-cell activation, because opsonization of Ags by complement components remarkably enhances both B cell activation (Carter and Fearon) as well as Ag uptake by B cells [6]. However, since IgM is unable to bind FcγRIIB, it fails to elicit negative feedback of humoral immunity. Thus, the imbalance of IgM and IgG in AID^−/−^ mice and humans could lead to unregulated B-cell proliferation, leading to the loss of peripheral tolerance. Other mechanisms, such as lack of high-affinity anti-idiotypic antibodies that might maintain B-cell homeostasis should also be considerable. Alternatively, insufficient elimination of external Ags due to the lack of class-switched and hypermutated Igs might drive B-cell activation. Elucidation of the precise mechanisms for unregulated B-cell proliferation as a result of AID deficiency is an important unresolved issue.

## Materials and Methods

### Reagents

Monoclonal Abs against B220 (RA3-6B2), CD3ε (145-2C11), CD8α (53-6.7), FoxP3 (FJK-16s) (eBioscience); CD4 (GK1.5), CD11b/Mac1 (M1/70), CD11c (HL3), CD16/CD32 (93), CD19 (1D3), CD25 (7D4), CD44 (IM7), CD62L (MEL14), CD69 (H1.2F3), CD95/Fas (DX2), CR1 (8C12), IL-17 (TC11-18H10.1) (BD Biosciences), IFN-γ (37895), IL-4 (30340) (R&D Systems) and polyclonal Abs against IgM (Southern Biotech) and CXCL13 (R&D Systems) were used. PNA was purchased from Vector Laboratories. Cy3-conjugated anti-rat IgG and Alexa488-conjugated anti-goat IgG secondary antibodies were purchased from Jackson Immunolaboratories and Invitrogen, respectively.

### Animal experiments

AID^−/−^ mice bred on a BALB/c background were kindly provided by Dr. T. Honjo [1], and maintained under SPF condition in RIKEN animal facilities. In certain experiments, mice were maintained under GF or CV condition at the animal facility of the University of Tokyo as described previously [34], [35]. To examine the role of T cells in gastritis development, 2.5–4×10^5^ CD3^+^CD4^+^ or CD3^+^CD8^+^ cells were isolated from the stomachs of 12-month-old AID^−/−^ mice with FACS Vantage SE (BD Biosciences), and were adoptively transferred to 2-month-old BALB/c^nu/nu^ mice (Clea Japan). Three months later, the stomachs of the recipient mice were excised for histological analysis. All animal experiments were approved by the Animal Research Committee of RIKEN Yokohama Research Institute and of the University of Tokyo.

### Histological analysis and immunofluorescence staining

For histological examination, 4% formalin-fixed, paraffin-embedded tissue sections were deparaffinized, rehydrated and stained with either hematoxylin-eosin or Alcian blue-hematoxylin. The specimens were analyzed for gastritis based on the criteria shown in Supplemental Table S1. Gastric-mucosa-infiltrating cells were analyzed by immunofluorescence staining. Frozen sections (5 µm) of gastric tissue were fixed with 4% Cytofix/Cytoperm (BD Pharmingen) and incubated with blocking buffer (Perkin Elmer) for 30 min at room temperature, followed by primary antibodies or isotype-matched control IgG. The binding of these antibodies was probed by fluorescent dye-conjugated secondary antibodies.

### Flow cytometric analysis

To flush out infiltrating cells, a 5% fetal bovine serum/phosphate-buffered saline (PBS) solution was injected into the gastric submucosa. The remaining gastric tissue was minced and subjected to collagenase digestion as described previously [35]. These treatments usually gave rise to a total of 1–6×10^7^ gastric infiltrating cells per mouse, dependent on the severity of inflammation. Splenocytes were prepared as described [36]. After Fc blocking, the samples were stained with fluorescent-dye- (FITC, PE, PECy7 or APC) or biotin-conjugated primary antibodies, followed by fluorescent-dye-conjugated streptavidin. Intracellular staining was performed with Cytofix/Cytoperm (BD) according to the manufacturer's instructions. The stained cells were analyzed with a FACS Calibur (BD).

### Cytokine analysis

Total RNA was extracted from gastric tissue using an RNeasy kit (QIAGEN), and was reverse-transcribed using ReverTra Ace-α (Toyobo). Q-PCR was performed to quantify cytokine mRNA expression levels using the SYBR®-Green PCR assay and the Thermal Cycler Dice Real Time System (Takara) essentially as described previously[35]. Protein concentrations of IFN-γ and IL-4 in culture supernatant of gastric CD4^+^ T cells were measured with a Cytometric Bead Array (CBA) kit (BD), and that of IL-17 was detected by an ELISA kit (R&D), following the manufacturers' instructions.

### Detection of autoantibodies

Gastric epithelia were obtained from AID^+/+^ mice maintained under GF condition, as described previously[37]. The prepared epithelia were homogenized in lysis buffer [150 mM NaCl, 20 mM Tris (pH 7.5), 1 mM EDTA, 0.1% Triton X-100] containing a protease inhibitor cocktail (Roche). The homogenate was centrifuged at 12,000 rpm at 4°C for 20 min to obtain soluble protein supernatants. To detect autoAb, 5 µg/ml of the gastric epithelial extract or porcine H^+^/K^+^ ATPase (Arotec Diagnostics) were immobilized on Maxisorp microtiter plates (Nunc). After blocking with 1% bovine serum albumin (BSA)/PBS, serum samples serially diluted with 1% BSA/1% casein/0.02% Tween 20/PBS [pH 7.4] were applied to the plate. Specific autoantibody binding was probed with a horseradish-peroxidase-conjugated anti-mouse IgM polyclonal Ab (Jackson ImmunoResearch Lab.), and was visualized with 3,3′,5,5′-tetramethylbenzidine and H_2_O_2_ as substrates.

### Statistical analysis

Data for all figures were collected from three or more independent experiments. Statistical significance of two groups was determined using the student's *t*-test. For comparison of three groups, the data were subjected to analysis of variance (ANOVA), followed by Fisher exact tests. The survival of mice of different genotypes was analyzed using the Kaplan-Meier method. Differences were considered significant at *P*<0.05.

## Supporting Information

Table S1 (0.12 MB PPT)

Figure S1 Analysis of TLOs observed in the gastric tissue of AID^−/−^ mice at 6 (A-B) or 12 (C) months of age. (A) Immunofluorescent staining for cell surface markers was performed using gastric samples from AID^−/−^ mice that contain TLOs. (B) High magnification of a gastric tissue section stained with H&E demonstrating migration of plasma cells (arrows) into the lamina propria. (C) TLOs were frequently coupled with type-A gastritis lesions in aged AID^−/−^ mice. Scale bars: 100 µm.
(12.91 MB PPT)

Figure S2 Total pathological score of gastritis development. The gastric tissue of AID^−/−^ and AID^+/+^ mice at different ages was analyzed by histology and scored following the criteria listed in Supplemental Table 1.
(0.19 MB PPT)
