# Supplementary material for: Activation-Induced Cytidine Deaminase Deficiency Causes Organ-Specific Autoimmune Disease
Source: PLoS One. 2008 Aug 21;3(8):e3033. doi: 10.1371/journal.pone.0003033 (PMC2515643; doi:10.1371/journal.pone.0003033)
Supplement: Table S1 — (0.12 MB PPT) [file pone.0003033.s001.ppt]

## Slide 1
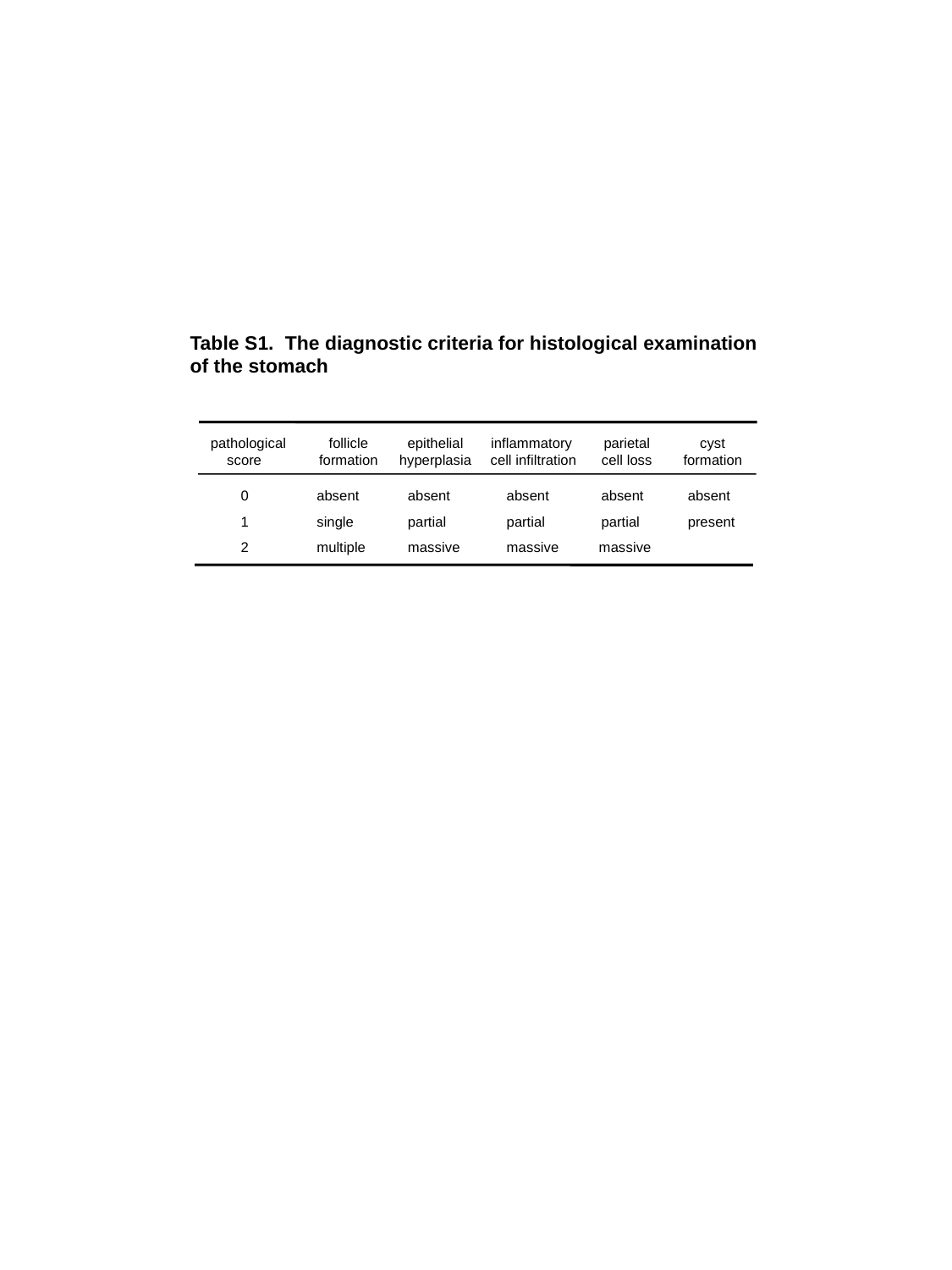

Table S1. The diagnostic criteria for histological examination of the stomach
pathological
 score
follicle
formation
epithelial
hyperplasia
inflammatory
cell infiltration
parietal
cell loss
cyst formation
0 absent	 absent 	 absent absent absent
1 single	 partial 	 partial partial present
2 multiple	 massive	 massive massive
