# Supplementary figures and images for: Activation-Induced Cytidine Deaminase Deficiency Causes Organ-Specific Autoimmune Disease
Source: PLoS One. 2008 Aug 21;3(8):e3033. doi: 10.1371/journal.pone.0003033 (PMC2515643; doi:10.1371/journal.pone.0003033)

## Slide 1
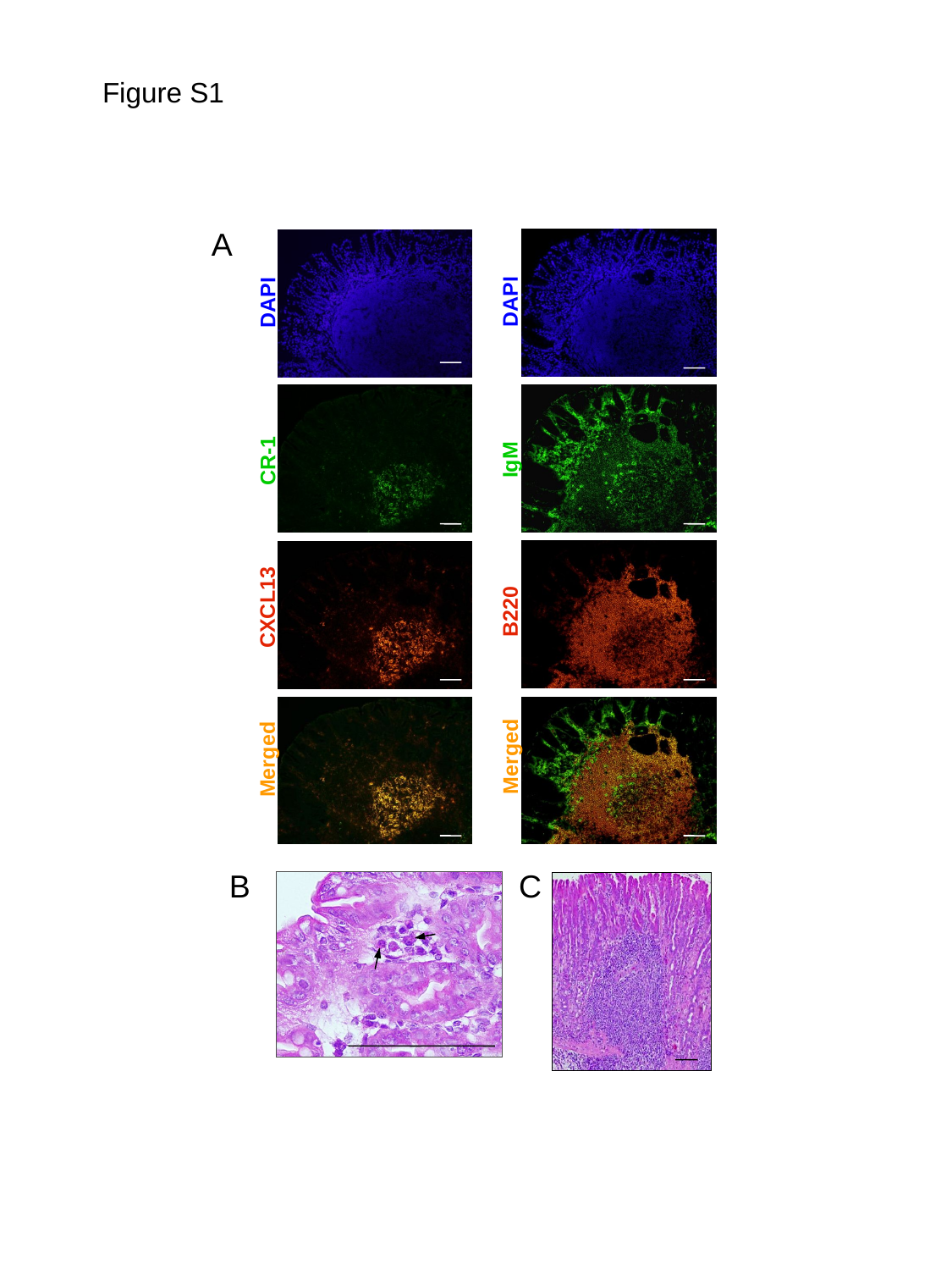

Figure S1
A
DAPI
DAPI
IgM
CR-1
CXCL13
B220
Merged
Merged
B
C

Supplement: Figure S1 — Analysis of TLOs observed in the gastric tissue of AID−/− mice at 6 (A-B) or 12 (C) months of age. (A) Immunofluorescent staining for cell surface markers was performed using gastric samples from AID−/− mice that contain TLOs. (B) High magnification of a gastric tissue section stained with H&E demonstrating migration of plasma cells (arrows) into the lamina propria. (C) TLOs were frequently coupled with type-A gastritis lesions in aged AID−/− mice. Scale bars: 100 µm. (12.91 MB PPT) [file pone.0003033.s002.ppt]
